# Baseline patterns of resting functional connectivity within posterior default-mode intranetwork associated with remission to antidepressants in major depressive disorder

**DOI:** 10.1016/j.nicl.2022.103230

**Published:** 2022-10-14

**Authors:** Yanxiang Ye, Chengyu Wang, Xiaofeng Lan, Weicheng Li, Ling Fu, Fan Zhang, Haiyan Liu, Kai Wu, Yanling Zhou, Yuping Ning

**Affiliations:** a The Affiliated Brain Hospital of Guangzhou Medical University, Guangzhou, China; b The First School of Clinical Medicine, Southern Medical University, Guangzhou, China; c Guangdong Engineering Technology Research Center for Translational Medicine of Metal Disorders, Guangzhou, China; d Department of Biomedical Engineering, School of Material Science and Engineering, South China University of Technology, China

## Abstract

**Background:**

The default mode network (DMN) is implicated in the pathophysiology of major depressive disorder (MDD), and functional connectivity (FC) involved in DMN is suggested to be associated with antidepressant remission. The goal of this study is to recognize relationships between FC within DMN and early amelioration in MDD patients and to further test the capacity of FC to predict early efficacy.

**Methods:**

In total 66 MDD patients and 57 healthy controls were recruited for resting-state functional magnetic resonance imaging scans at baseline. After four weeks of treatment with Escitalopram or Venlafaxine, patients were divided into subgroups with remitters (R, n = 31) and non-remitters (NR, n = 35). Independent component analysis (ICA) was used to compare intranetwork functional connectivity (intra-FC) in DMN between the three groups.

**Results:**

Relative to NR-MDD group and HCs, the R-MDD group showed significantly higher intra-FC in the right angular gyrus of DMN, and the intra-FC was positively correlated with the reduction ratio of the depressive symptom scores. The ROC curve analysis revealed that intra-FC exhibited a high diagnostic value for remission.

**Conclusion:**

These findings indicated that intra-FC related to the DMN is a prognostic marker that can potentially predict early remission of symptoms after antidepressant treatment.

## Highlights

• We adopted ICA to investigate baseline intranetwork FC that characterizes remission to antidepressant.
• Baseline higher intranetwork FC between the right angular and pDMN was associated with early improvement following 4 weeks antidepressant medications.
• Significant correlation and ROC curve analysis revealed a high diagnostic value for prediction of early antidepressant efficacy.

## 1 Introduction

Major depressive disorder (MDD) is a widespread mental disease along with increasing incidence rate, disability, and suicide rate, characterized by emotional, cognitive, and behavioral impairments. Nowadays, antidepressants are still steadily recommended as the first-line treatment for MDD patients. Nevertheless, about two-thirds of patients with depression failed to benefit from monotherapy, which not only prolongs recovery time and consequential suffering of patients, but also enormously increases the burden and waste of medical resources (Anthes, 2014, Bauer et al., 2015, Qaseem et al., 2016). According to the antidepressant treatment guidelines, clinicians need 4–6 weeks to evaluate the treatment response and determine whether to change the treatment regimen, which prolongs patient suffering and increases the waste of medical resources (Bauer et al., 2015, Qaseem et al., 2016). Owing to the insufficient evidence of biological mechanisms and measures that enable clinicians to assess the efficacy of treatments in advance and anticipate clinical cures, it’s a hot topic clinically to identify biomarkers that can predict the response and remission of MDD patients before pharmacological treatment.

Although consistent conclusions to distinguish effective remission early from non-remission in individualized medicine usage remain unclear, functional magnetic resonance imaging (fMRI) has proven to be an important tool in elucidating the pathophysiological mechanisms of MDD and has made important breakthroughs in understanding the neural basis of MDD. A meta-analysis of antidepressant response biomarkers revealed that imaging biomarkers showed the greatest effect sizes compared to other approaches (Voegeli et al., 2017), which indicate better predictive performance for imaging biomarkers. Regarding the rapid advances in neuroimaging techniques of the brain, to date, intensive researches with neuroimaging techniques suggest that alterations in several functional brain networks (FBNs) play an important role in mood regulation (Brakowski et al., 2017), and accordingly, the prediction for symptoms improvement after antidepressant treatment (Hou et al., 2018), which subserves shedding light on the biological mechanisms of depression.

Among different FBNs, the default-mode network (DMN) is widely believed to be implicated in MDD patients’ pathopsychological characteristics, e.g. self-referential processing, rumination, automated information processing (Ho et al., 2015, Nejad et al., 2013, Vatansever et al., 2017). Numerous studies have explored different functional connectivity (FC) of the DMN between MDD patients and healthy controls (Li et al., 2013, Peng et al., 2021, Yan et al., 2019), and pre-treatment functional connectivity associated with response to pharmaceutical and non-pharmaceutical treatments has been found (Dunlop et al., 2017, Goldstein-Piekarski et al., 2018, Salomons et al., 2014). In addition, the DMN has been hypothesized to be centrally implicated in the treatment response of antidepressant medications at the neural mood circuitry, which is entailed by numerous neurotransmitter systems including serotonin, dopamine, gamma-aminobutyric acid, etc (Conio et al., 2020, Hahn et al., 2012, Northoff et al., 2007). Correspondingly, these neurotransmitters are recognized neurobiological targets for antidepressant medications, and their association with MDD pathophysiology is substantiated and well documented in medical literatures (Duman et al., 2019, Fakhoury, 2016, Hirschfeld, 2000). Previous studies have borne out claims that antidepressants, consisting of both serotonin reuptake inhibitors (SSRIs) and serotonin norepinephrine reuptake inhibitors (SNRIs), might normalize anomalous FBNs between the frontal and limbic regions (Brakowski et al., 2017, Dichter et al., 2015, McCabe et al., 2011, Vai et al., 2016) via fMRI analyses of different types, with possibly pivotal role of the posterior DMN (Li et al., 2013, Posner et al., 2013). For example, identified connectivity patterns within the DMN are regarded as an important network in predicting response to sertraline (Kaiser et al., 2015). Inference could be drawn that functional connectivity associated with DMN is a prognostic marker to distinguish remitters with major depressive disorder (R-MDD) from non-remitters with major depressive disorder (NR-MDD) (Cui et al., 2021, Martens et al., 2021, Spies et al., 2017). Consequently, we hypothesized that the pretreatments FC linked to the DMN of R-MDD group and NR-MDD group would be different from each other and also relative to healthy controls (HCs).

Apart from a seed-based method, an alternative method based on data-driven parcellation is frequently applied to analyze and process fMRI data, e.g. independent component analysis (ICA). ICA is a commonly used blind source separation method that attempts to decompose a mixed signal into independent components (ICs) without prior knowledge of the ICs structure to analyze and process fMRI data, while seed-based method have some restrictions for explorative analysis because it usually requires a predefined hypothesis as to the role of a region with a priori selection, and a choice of a ‘seed’ – region of interest (ROI) can be quite subjective with slightly different seeds giving different results. By extracting statistically optimal independent components without a priori selection, ICA is considered to be a successful method to identify typical resting-state fMRI of spatially independent FBNs, such as visual networks, auditory networks, executive control networks along with, most importantly, the DMN (Calhoun et al., 2008, Martens et al., 2021, Spies et al., 2017).

In a nutshell, the purpose of this study was to use the data-driven method to compare pretreatment differences in baseline intranetwork functional connectivity (intra-FC) within the DMN between R-MDD and NR-MDD.

## 2 Materials and methods

### 2.1 Participants

The present study was carried out following the latest version of the Declaration of Helsinki and supported by ethics committees of the Affiliated Brain Hospital of Guangzhou Medical University (reference number 030/2016). MDD patients between the ages of 18–65 years were drawn from a precision medicine study on the treatment of depression using Venlafaxine and Escitalopram (clinical trial number: ChiCTR1800017626). After studying procedures were fully explained to participants, they offered written informed consent to take part in the study. Inclusion criteria for all the patients were as follows: (1) fulfilled the criteria for MDD according to the Diagnostic and Statistical Manual of Mental Disorders (Fifth Edition) (DSM-5); (2) all the MDD groups never took antidepressants before, or were washed off medications due to patients’ subjective or irresistible reasons for at least 4 weeks prior to trial inclusion; (3) without any contraindications for MRI examination; (4) the Hamilton Depression Rating Scale-17 (HAMD-17) scores ≥ 17. Exclusion criteria were: (1) subjects with a history of other major psychiatric disorders that met the criteria of axis I of the Diagnostic and Statistical Manual of Mental Disorders, 5th ed, had current serious and unstable somatic diseases or a history of neurologic or other chronic medical conditions; (2) women were either breastfeeding or pregnant; (3) subjects were unable to comprehend and finish neurocognitive assessments. Finally, 66 MDD patients completed both baseline fMRI scan and a 4-week period flexibly dosed, open-label antidepressant treatments schema, including either a selective serotonin reuptake inhibitor - Escitalopram or a serotonin-noradrenaline reuptake inhibitor - Venlafaxine prescribed by treating clinicians according to patients’ conditions and clinically recommended dose ranges. Both doses of antidepressants were converted to the equivalent dose of Fluoxetine as covariates for statistic analysis based on the conversion formula (Fluoxetine 20 mg = Escitalopram 9 mg = Venlafaxine 74.7 mg) (Furukawa et al., 2019).

The healthy participants met the inclusion criteria as (1) age between 18 and 65 years old; (2) no psychiatric disorders have been diagnosed before; (3) without chronic and somatic diseases; (4) no contraindications for magnetic resonance scanning such as metal implants and claustrophobia. A total of 57 healthy controls were recruited from the local community and completed the baseline fMRI scan (Table 1 for demographic information).

**Table 1:** Demographic and clinical characteristics of subjects.

Variables	R-MDD	NR-MDD	HC	T/F/Z/χ^2^	P
Numbers of subjects	31	35	57	–	–
Age	29.77 ± 10.54	25.83 ± 8.14	30.51 ± 7.76	3.355	**0.038**^a^
Gender (male/female)	13/18	12/23	26/31	1.150	0.563^b^
Education (years)	12.13 ± 3.16	13.29 ± 3.04	–	−1.513	0.135^c^
Course of disease (months)	29.15 ± 28.01	26.67 ± 27.45	–	0.362	0.719^c^
First-episode of depression (yes/no)	16/15	19/16	–	0.047	0.828^b^
Age at onset(years)	27.45 ± 10.53	23.71 ± 8.22		−1.630	0.103^d^
Heavy alcohol users(yes/no)	2/29	3/32		–	0.558^e^
Baseline HAMD-17	22.39 ± 4.37	23.74 ± 4.92	–	−1.128	0.259^d^
Baseline HAMA-14	17.00 ± 6.23	23.11 ± 5.97		−4.069	**0.001**^c^
Antidepressant regimen(Escitalopram/Venlafaxine)	16/15	14/21		0.894	0.344^b^
Fluoxetine equivalent dosage regimen(mg)	27.74 ± 12.96	31.29 ± 13.68		−1.039	0.299^d^

Abbreviations: R, remitters; NR, Non-remitters; MDD, major depressive disorder; HC, healthy controls; HAMD-17, 17-item Hamilton Depression Rating Scale; HAMA-14, Hamilton Anxiety Scale.

a One-way ANOVA.

b Chi-square test.

c Two-sample *t*-test.

d Mann-Whitney U.

e Fisher’s exact test.

In total 125 MDD patients and 57 HCs were recruited. In the remaining 59 participants, relevant data were not available at the end of the treatment period (thirty-nine patients dropped out before the 4-week assessment, seventeen patients’ images were unavailable due to excessive motion, and three patients were not scanned due to safety concerns revealed at the scanning appointment).

### 2.2 Assessment

HAMD-17 and Hamilton Anxiety Rating Scale-14 (HAMA-14) were both contained in the clinical assessment before and after 4-week antidepressant treatment to evaluate participants’ depressive and anxious symptom degrees. Additionally, participants’ age, gender, education, age of onset of depression, antidepressant regimen, and Fluoxetine equivalent dosage regimen were recorded for analyses. Clinically, the operational criteria for patients with depression to achieve clinical remission are as follows: HAMD-17 (Hamilton Depression Scale-17) scores ≤ 7, and no longer meet the DSM diagnostic criteria for depression (Zimmerman et al., 2013). Thus, according to the most widely used standard for clinical remission of depression, a HAMD-17 of ≤ 7 was used to define remission at week 4, then we divided MDD patients into two different subgroups of remitters (R-MDD) and non-remitters (NR-MDD) by the cutoff point 7 after week 4 medication treatment, so as to ensure that clinical symptoms of patients were reduced and patients can reach the ‘remission state’ (Association, 2000). Additionally, aiming to perform the magnitude of the antidepressant response, we calculated the reductive ratio in HAMD/HAMA scores from baseline to the end of the antidepressant treatment period (reductive ratio = [total score before treatment – total score after treatment] / total score before treatment) (Drysdale et al., 2017). All clinical assessors were psychiatrists with either master or doctor’s degrees, who attended systematic training sessions like reviewing a teaching video for assessment and reached an intra-class correlation coefficient > 0.9 for interrater reliability testing before the start of the study.

### 2.3 Image processing of rs-fMRI

The fMRI data was acquired at baseline in the Affiliated Brain Hospital of Guangzhou Medical University (3.0-Tesla Philips Achieva scanner). Using a 3D distortion gradient-echo sequence (repetition time 8.2 ms, echo time 3.8 ms, inversion time 1100 ms, flip angle (FA) = 8°, 188 slices, slice thickness 1 mm, gap 0 mm, field of view 256 × 256, inversion time 0) to obtain a high-resolution T1 weight image. The 8-minute rs-fMRI scan datasets of the whole brain were acquired with a gradient-echo echoplanar imaging (GRE-*EPI*) sequence with the following detailed parameters: repetition time (TR) = 2000 ms, echo time (TE) = 30 ms, flip angle (FA) = 90°, slice thickness = 4 mm, number of slices = 33, and field of view (FOV) = 220 × 220 mm. Subjects were informed to lay still, relax and stay awake with their eyes closed during the fMRI scan.

### 2.4 Resting-state fMRI preprocessing

Image preprocessing was carried out using tools from Data Processing Assistant for Resting-State fMRI 6.0 (DPASF 6.0) running in MATLAB R2019b (The Mathworks, Natick, MA, USA) (Yan et al., 2016). For all images, the first 10 functional volumes were removed to allow for signal stabilization, and the remaining 230 volumes underwent correction for slice acquisition timing and head movement realignment where mean framewise displacement (FD) based on the Jenkinson model (FD-Jenkinson) was computed by averaging the FD from every time point for each participant (Jenkinson et al., 2002). The fMRI images exhibiting excessive head motion (translation > 3 mm or rotation > 3°) were discarded to eliminate the influence of machine motion and scanning for further analyses.

The standard Montreal Neurological Institute Echo-planar Imaging (*EPI*) template is used to perform spatial normalization processing on the motion-corrected functional image (resampled to 3 × 3 × 3 mm^3^). In addition, the images were spatially smoothed with a 6-mm full width at half maximum (FWHM) Gaussian kernel.

### 2.5 Functional connectivity analyses

On the basis of the preprocessed rs-fMRI data, spatial independent component analysis (ICA) was conducted for all 123 participants with the Informix algorithm (Bell and Sejnowski, 1995) repeating 100 times to ensure the reliability of stable components in the Group ICA of fMRI Toolbox (GIFT) software package (mialab.mrn.org/software/gift), which is used to decompose the rs-fMRI image into a series of independent spatial patterns (Jafri et al., 2008). The number of independent components (N = 38) was estimated automatically by the software using the method of minimum cluster analysis (Liao et al., 2010). Afterward, the averaged IC was applied for each subject for ICA separation. Each data set was disposed to reduce its dimensions via principal component analysis using two reduction steps. The spatial graphics and time series of independent components that best matched the template of each subject were transformed into z-values to represent the contribution values of components. Then the results of DMN were checked and selected visually referring comprehensively to the DMN template provided by the previous literatures (Arbabshirani et al., 2013, Yeo et al., 2011) for subsequent statistical processing. Finally, the GICA back reconstruction method is used to obtain the participant-specific spatial map and time courses.

### 2.6 Statistical analyses

Clinical and demographic characteristics were analyzed by Statistical Package for the Social Sciences version 25.0 (SPSS 25.0; IBM, Armonk, NY, USA). According to the type of distribution and the size of the data, we perform *t*-test, one-way analysis of variance or χ2 test to compare the differences between different groups. Statistics were reported in the form of mean, standard deviation, or frequency.

The group analysis of the imaging data was performed with Statistical Parametric Mapping software (SPM12, http://www.fil.ion.ucl.ac.uk/spm). To identify the DMN, for each selected IC, z-FC were analyzed in every group respectively for a one-sample *t*-test, corrected via Family wise error (FWE) multiple comparisons with a voxel-level p < 0.05. To detect group differences in intra-FC, analysis of variance (ANOVA) was applied to detect group differences in intra-FC to analyze the mean z-maps of DMN within the explicit mask created by merging the results of the aforementioned one-sample *t*-test of each group, within the overall mask calculated by merging the images of the aforementioned one-sample *t*-test of three groups, and gender, age, frame wise displacement (FD), baseline HAMA scores were used as covariates. Moreover, family wise error (FWE) cluster-corrected, cluster-level p < 0.05 was used for the correction of multiple comparisons in second-level analyses with a primary voxel-level threshold of p < 0.001. Covered by the explicit mask that was extracted from regions of F contrast differences and corrected via the same FWE-cluster multiple comparison correction method (p < 0.001 at the initial voxel level and p < 0.05 at the cluster level), the post-hoc statistical differences were analyzed. BrainNet Viewer software (https://www.nitrc.org/projects/bnv) was used to plot the marked brain regions.

Meanwhile, zFC data were extracted from brain regions that were significantly different between the three groups and post hoc test was performed between each pair of groups (P < 0.05, Bonferroni corrected) in SPSS software and illustrated in the form of mean and standard deviation to corroborate the results of marked brain region showing difference illustrated from SPM. One-way analysis of covariance (ANCOVA) was applied to compare the differences in Z values among the R, NR, and HC groups in the FC analysis by SPSS. Age, gender, framewise displacement, baseline HAMA scores were all involved as covariates in the statistical analysis.

Additionally, to explore the remitted mechanisms of clinical symptoms associated with MDD default network dysfunction, partial correlation analysis was performed to indicate the relationships between the baseline zFC levels of abnormal DMN and clinical traits (including HAMD-17 reductive ratio, HAMA-14 reductive ratio) in the MDD patient group by controlling the confounding factors age, gender, Fluoxetine equivalent dosage and baseline HAMA scores (only for HAMD-17 reductive ratio) in pooled MDD patients (including both the R-MDD and NR-MDD groups).

Lastly, receiver operating characteristic (ROC) curves were carried out to illustrate the possible predictive value of intranetwork FC that showed significant differences between R-MDD and NR-MDD groups. Sensitivity and specificity were estimated via zFC values to distinguish R-MDD and NR-MDD, and the maximum Youden index (Youden index = sensitivity + specificity − 1) (Youden, 1950) was calculated to determine the zFC of optimum level that generated the top combination of sensitivity and specificity, as had been explored in earlier researches(Xiao et al., 2021, Zhang et al., 2022). The area under the curve (AUC) was performed to evaluate the likelihood of classifying the different MDD groups. Nevertheless, these assessments of the predicted values are aimed at reflecting the classification ability of the identified areas in the sample, rather than independent verification.

## 3 Results

### 3.1 Demographics and clinical features

Table 1 summarizes the demographic details and clinical characteristics of the participants in the study. The three groups were well matched for gender. However, the mean age of the NR-MDD group was slightly, but significantly, smaller than the other two groups and showed significant differences (F = 3.355, p = 0.038). Furthermore, we found no significant differences in gender, education level, course of disease, first episode of depression, age at onset, heavy alcohol users(yes/no), baseline HAMD-17, antidepressant regimen, Fluoxetine equivalent dosage regimen between the R-MDD group and NR-MDD group (all P > 0.05), while NR-MDD group showed higher levels of anxiety according to baseline HAMA-14 compared with the R-MDD group (F = −4.069, p < 0.001).

### 3.2 DMN selection

**Fig. 1:**
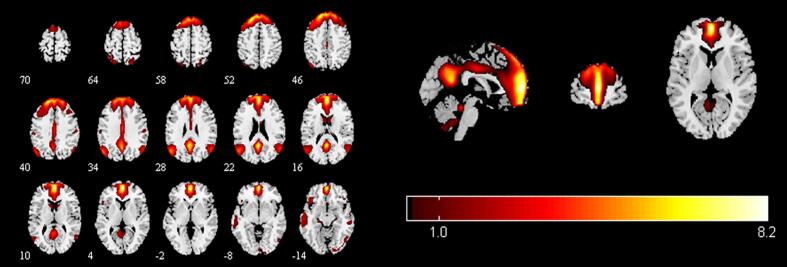

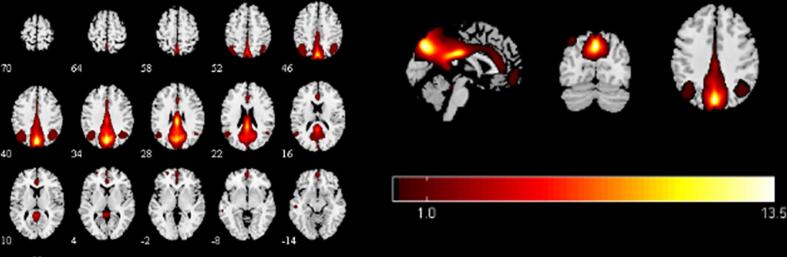

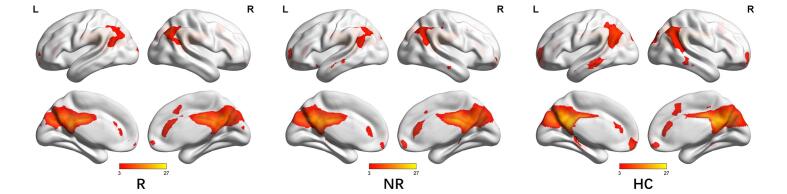

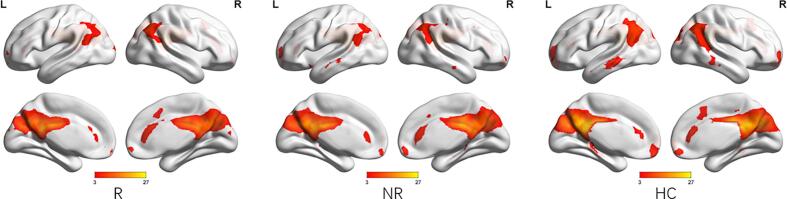
Images showing the DMN extracted by independent component analyses in all subjects. (a)The IC identified as aDMN. (b)The IC identified as pDMN. (c)The spatial patterns of aDMN intrinsic connectivity networks respectively in R-MDD, NR-MDD and HC groups using one-sample t-tests. (d)The spatial patterns of pDMN intrinsic connectivity networks in R-MDD, NR-MDD and HC groups using one-sample t-tests. The color scale represents *t*-values in each network (p < 0.05, FWE Voxel-level corrected). Abbreviations: IC, independent component; aDMN, default-mode network; pDMN, posterior default mode networks; R, remitters; NR, Non-remitters; MDD, major depressive disorder; HC, healthy controls.

To identify meaningful ICs, each IC was further visually checked to guarantee all the FBNs are in grey matter and have low spatial overlap with white matter structures, ventricles, and vascular. Out of 38 ICs extracted from the spatial concatenation, 10 meaningful ICs were identified as functional networks that had peak activations in gray matter, and exhibited primarily low-frequency power and low spatial overlap with white matter structures, ventricles, and vascular, while the other 28 components (see Table S1 and Fig. S1-Fig.S28 in the Supplementary Material) represented physiological noise (arteries, veins and CSF), head motion and magnetic susceptibility artifacts. Subsequently, two ICs were visually checked and recognized as anterior and posterior default mode networks (aDMN and pDMN) by artificial selection. Figures of ICs comprising aDMN and pDMN are displayed in Fig. 1A andB.

### 3.3 Statistic comparison of intra-FC values differences between groups in DMN

**Fig. 2:**
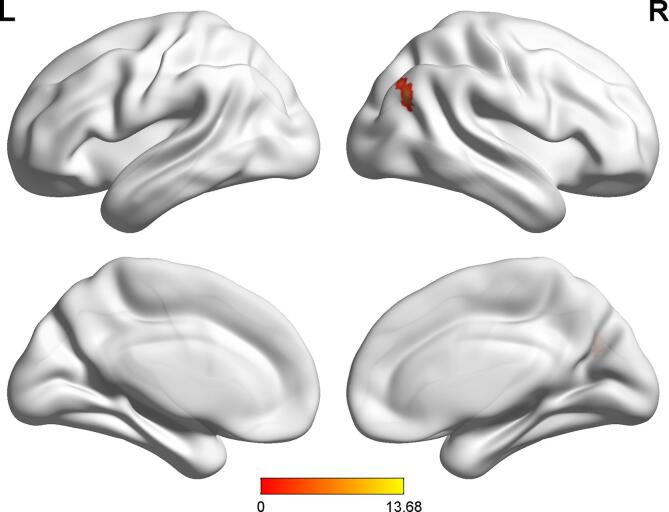

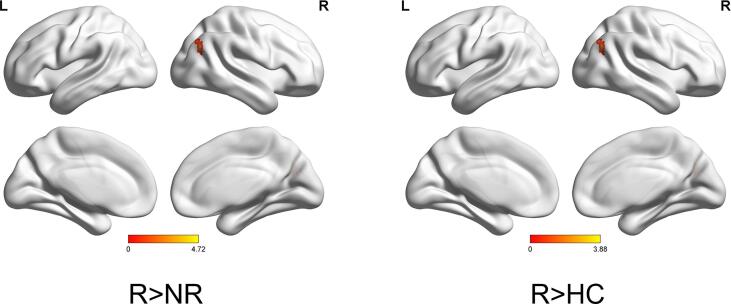
(a)Brain region of F contrast differences with the right angular of pDMN intranetwork between the three groups, Multiple comparison correction were used with a family-wise error cluster-level correction, voxel- level of p < 0.001, cluster-level p < 0.05. (b)Red colors indicated the increased intranetwork functional connectivity FC between the default mode network and right angular gyrus in the R group compared with the NR group and HC group, as shown by BrainNet Viewer. n.s., not significant. (For interpretation of the references to color in this figure legend, the reader is referred to the web version of this article.)

**Fig. 3:**
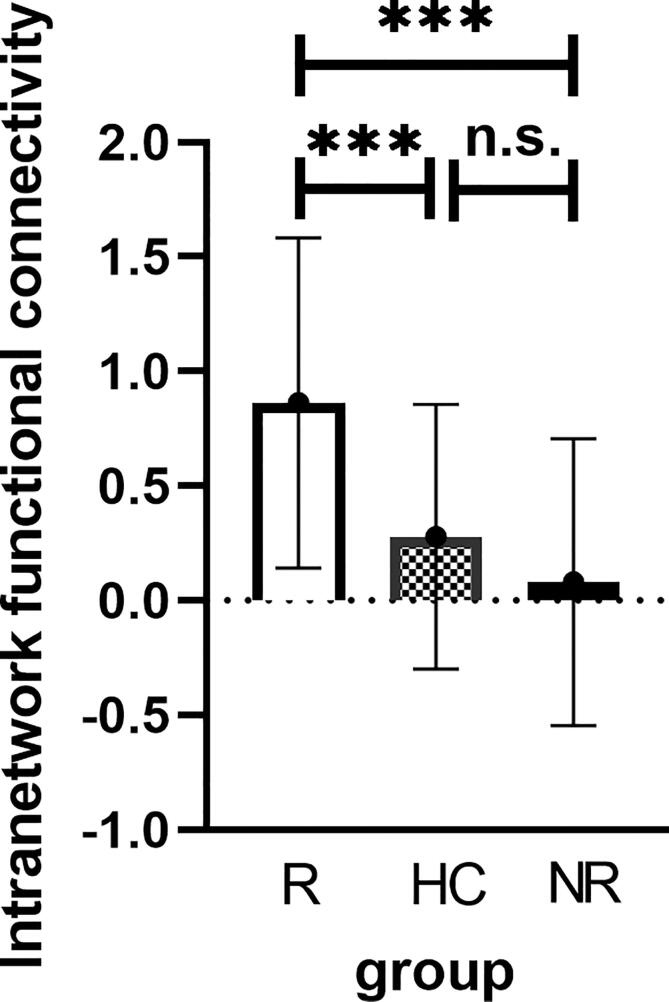
Significant mean zFC value differences extracted from right angular gyrus for each pair group (R vs NR, R vs HC, and R vs HC) between the default mode network and right angular gyrus/parietal gyrus. One-Way ANCOVA, F = 14.09, Post-hoc, Bofferoni corrected, p < 0.05, two-tail. Error bars denote standard deviation. Abbreviations: R, remitters; NR, Non-remitters; ANOVA, analysis of variance. n.s., not significant. ***p < 0.001.

Corrected by one-sample t-tests, positive values revealed the spatial pattern of the aDMN and pDMN in the MDD patients and healthy control subjects (Fig. 1C, Fig. 1D). After adjusting for age, gender, baseline HAMA scores and FD with FWE cluster-level correction, ANOVA analysis in SPM revealed significant group differences (FWE cluster-level correction, voxel-level of p < 0.001, cluster-level p < 0.05, cluster size ≥ 21) in baseline intranetwork functional connectivity with the right angular gyrus (AG) of pDMN (Fig. 2A and Table 2) between (1) R-MDD and NR-MDD (FWE cluster-level correction, voxel-level of p < 0.001, cluster-level p < 0.05, cluster size ≥ 19), (2) R-MDD and HC (FWE cluster-level correction, voxel-level of p < 0.001, cluster-level p < 0.05, cluster size ≥ 11) (Fig. 2B and Table 2), whereas group differences in baseline intranetwork functional connectivity of aDMN were not detected. Simultaneous0ly, One-way ANCOVA was used to compare the differences of zFC among the R, NR and HC groups in the pDMN analysis via SPSS, and results showed that differences were compatible with SPM graph presentation (Fig. 3), where the zFC of the pDMN was increased in the R-MDD compared to the NR-MDD and HCs.

**Table 2:** Brain areas with significant differences in the intra-FC values among three groups.

CONTRAST	Network	Cluster	MNI Coordinates			K(cluster size, number of voxel)	Peak F/*t*-value
			x	y	z		
F contrast	pDMN,	Right angular gyrus	42	−66	33	21	13.68
R＞NR	pDMN,	Right angular gyrus	42	−66	30	19	4.72
R＞HC	pDMN,	Right angular gyrus	42	−66	27	11	3.88

Abbreviations: R, responders; NR, non-responders; HC, healthy controls; pDMN, posterior default mode network.

Of note, after performing two sample *t*-test in SPM, we found no significant difference in FC with AG between MDD (pooled R and NR) and HC group.

### 3.4 Intra-FC changes in MDD patients relate to HAMD-17 and HAMA-14 scores

**Fig. 4:**
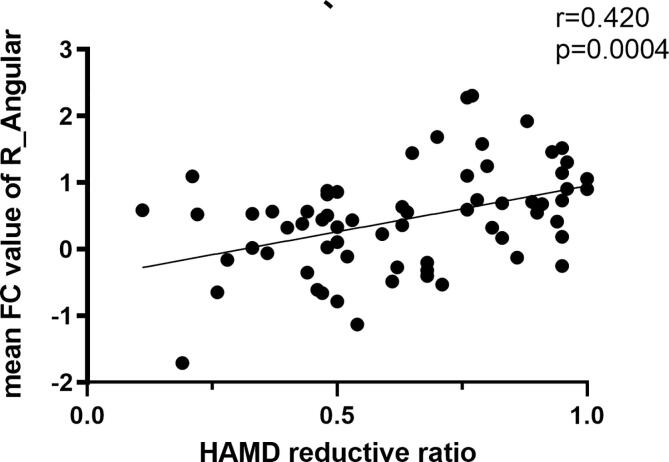

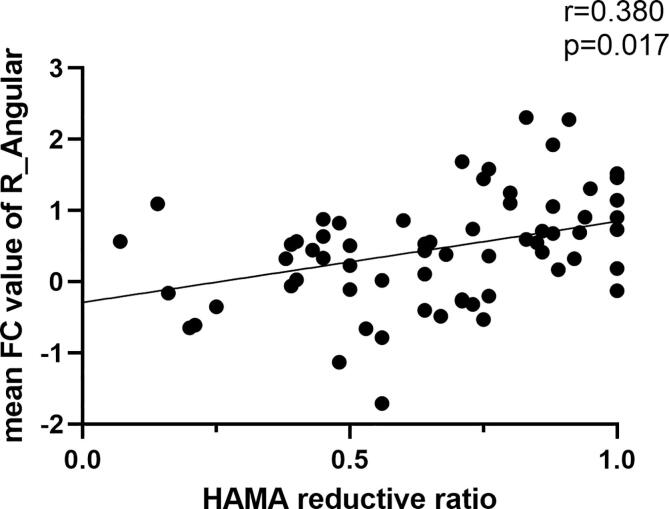
**A.** The HAMD reductive ratio showed a positive correlation with the intranetwork FC values of the right angular (r = 0.420, P = 0.0004); Fig. 4**B**. The HAMA reductive ratio showed a positive correlation with the intranetwork FC values of the right angular (r = 0.380, P = 0.0017).

The partial correlation analyses revealed that RSFC extracted from right angular among DMN network was positively associated with HAMD-17 reductive ratio (r = 0.420, p = 0.0004) (Fig. 4A), and with HAMD-14 reductive ratio (r = 0.380, p = 0.017) (Fig. 4B). Variables including age, gender, and years in education were involved as covariates in statistical analysis.

### 3.5 ROC curve analysis

**Fig. 5:**
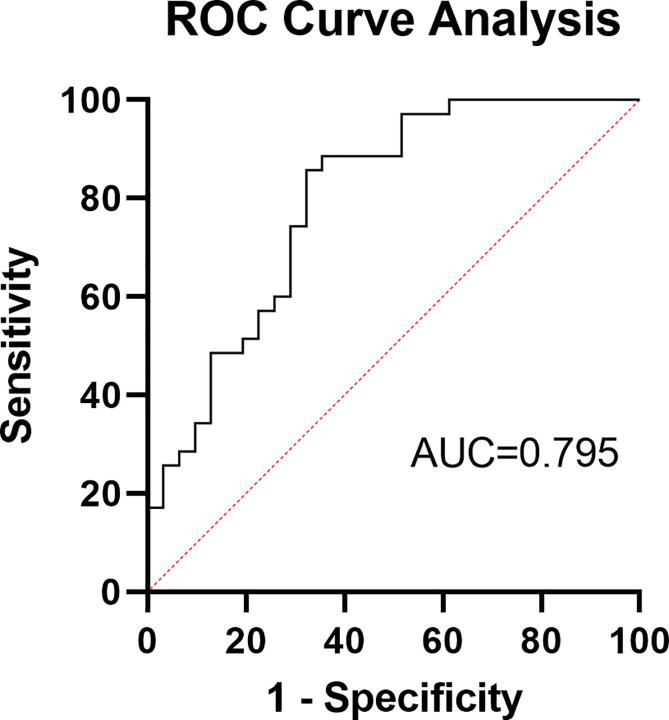
ROC curves for differentiating the R-MDD group and the NR-MDD group. Abbreviations: ROC, receiver operating characteristic; AUC, area under the curve.

As shown in receiver operating characteristic curves (ROC) analysis (Fig. 5), the AUC value of FC between the right angular and DMN for differentiating the R-MDD group from the NR-MDD group was 0.795, 95 %CI (0.678, 0.885), p < 0.0001, Youden index J was 0.5346, and optimal cut-off FC score was 0.589 with a sensitivity of 85.71 and specificity of 67.74.

## 4 Discussions

Here, we adopted ICA, a data-driven approach, rather than seed-based one, to investigate baseline intra-FC of pDMN that characterize remission to antidepressant medications prior to treatment in 66 MDD patients and 57 healthy controls. Our study not only allowed for a relatively unbiased exploration of FBNs, but also reduced the amount of computation in the meanwhile to separate the ICs. By using the methodology of ICA, our study suggested possibilities that increasing intra-FC within DMN tended to discriminate between patients with remitters and non-remitters following four weeks of antidepressant treatment, irrespective of medication type.

Korgaonkar et al. (2020) reported a whole-brain functional connectivity analysis to identify connectomic predictors of antidepressant treatment response and demonstrated that remitters were distinguished from non-remitters by greater connectivity within the DMN. Previous research also revealed that the treatment-resistant depression (TRD) group was more likely to perform a more decreased FC than the treatment-sensitive depression (TSD) group, generally in connected regions within DMN (precuneus, angular gyrus, and inferior parietal lobule) (Guo et al., 2013), which is consistent with our results.

DMN consists of a series of brain regions, such as the medial prefrontal cortex, posterior cingulate cortex /precuneus, and inferior parietal regions/angular gyrus (Lim et al., 2014), and these brain regions clearly prove an accordant pattern of deactivation during the initiation of task-related activity (Andrews-Hanna et al., 2010, Greicius et al., 2003, Raichle et al., 2001). In general, as a part of DMN, the angular gyrus is responsible for semantic processing, helps connect perception, attention, spatial cognition and action, and recalls the memory of episodes, which can be impaired in MDD (Klumpp and Deldin, 2010). Changes in the FC of the DMN are implicated in the cognitive control functions, self-referential processing, and emotional activity, and strong evidence supports that disruptions in DMN could be the pathophysiologic cause of cognitive and mental dysfunction in MDD patients (Greicius et al., 2007, Raichle, 2015).

Abnormal FC between the default mode network and right angular gyrus/inferior parietal lobule in responders compared with healthy controls has been detected as well in MDD patients, and results revealed that antidepressants treatment remission was associated with reduced FC of the pretreatment posterior DMN (Martens et al., 2021). Interestingly, our finding contrasts with this previous study and suggests that the elevated FC of right AG within pDMN may be a target for rapid antidepressant effect. This discrepancy could be attributed to multifold reasons, for instance, we have large sample size (66 patients and 57 HCs vs 34 patients and 31 HCs), different follow-up period (4 weeks vs 6 weeks), software tool usage (SPM vs FSL), multiple comparison correction (parameter analysis vs nonparametric permutation analysis), and our treatment consists of two antidepressants instead of monotherapy, etc. Further research will be needed to elucidate the reasons for the observed inconsistency.

Furthermore, we observed that the intra-FC values of the right angular showed a positive correlation with the HAMD reductive ratio and the HAMA reductive ratio. It can be implied from the results that MDD patients with greater than normal connectivity, particularly related to the pDMN, were the most likely to benefit from antidepressant treatment and to achieve acute depressive remission, as well as concomitant symptoms such as anxiety. On the other hand, clinically this could be extrapolated that MDD patients with an anomalously reduced functional connectivity from pDMN are less likely to benefit acutely from antidepressant medications as first-line treatments. Congruent with previous studies (Broyd et al., 2009, Hamilton et al., 2011, Holtzheimer and Mayberg, 2011), Wu et al. (2020) assumed that baseline hyperconnectivity within the DMN predicted larger responses and account for the maladaptive depressive rumination and the excessive self-focus processing. Chin et al. noted that higher connectivity within the DMN predicted better antidepressant efficacy for Sertraline (Chin et al., 2020), suggesting MDD patients are associated with impaired neuronal plasticity, while brain-derived neurotrophic factor (BDNF) could play a key role in the modulation of neuronal networks (Lee and Kim, 2010). Analogous to BDNF, increasing DMN intra-FC more or less represents a neurotrophic capacity that reserves plasticity of neuronal networks so that it enables antidepressants to normalize anomalous DMN in MDD patients. A meta-analysis indicated hyperconnectivity within the DMN in MDD patients compared with healthy control subjects (Kaiser et al., 2015b), while patients with treatment-resistant major depression who showed increased within-network connectivity of the DMN than healthy control subjects exhibited decreased DMN connectivity after repetitive transcranial magnetic stimulation treatment (Kaiser et al., 2015). Similarly, the association of increased intra-FC of the pDMN with better remission to Escitalopram and Venlafaxine suggests that improvement of neural dysfunction in MDD as compared with HCs may be a potential mechanism of antidepressants. Thus, we contend that clinical remission may actually require intact or higher than normal pre-treatment DMN intra-network functional connectivity, rather than indicate the alteration from abnormal to normal connectivity.

Nevertheless, it remains unclear whether other monotherapy or conjunction with antidepressants with a longer follow-up periodperiodperiod (e.g. 6–8 weeks) could result in the same consequence, which requires further investigations for verification, notwithstanding a general response associated with FC biomarker to 3 antidepressants (Escitalopram, Sertraline, and Venlafaxine-XR) has been found (Korgaonkar et al., 2020).

## 5 Limitations

There are several limitations of the study that should be addressed. Firstly, the cross-sectional state of this study limits its scope to a better understanding of time impact, and longitudinal studies help to determine whether FC changes in the right angular gyrus after treatment. Secondly, the sample size of this study was relatively small, which reduces the power and increases the likelihood of type 2 error. Thirdly, although we transform the two kinds of antidepressants into the equivalent dose of Fluoxetine, the truth is that the underlying mechanism of the two antidepressants differs relatively, which caused mixed effect of two different antidepressants, and further investigation is required to confirm the monotherapy consequence. Fourthly, although the mean age of the R-MDD group was above that of the NR-MDD group, they were both representative of the adult population with MDD. Fifthly, the criterion for clinical remission of depression is HAMD-17 ≤ 7, which is based on symptomatic judgment. We supposed that depressive rehabilitation including recovery of psychosocial function and experience of patients' subjective feelings should be taken into consideration. Future work with multiple and complementary assessments in the evaluation of the therapeutic response should address this question. Last but not least, we only explored the default network, other depression-related networks e.g. salience network and frontoparietal network need further investigation.

## 6 Conclusion

Taken together, our findings identified that baseline higher intranetwork FC, especially between the right angular and pDMN, was associated with early improvement following 4 weeks of antidepressant medications. Higher than normal FC within pDMN could be a prerequisite mechanism for remission on antidepressant treatments and potentially used to assist in the prediction of early antidepressant efficacy

## CRediT authorship contribution statement

**Yanxiang Ye:** Investigation, Formal analysis, Writing – original draft, Visualization. **Chengyu Wang:** Validation, Methodology, Investigation. **Xiaofeng Lan:** Validation, Methodology, Investigation. **Weicheng Li:** Validation, Methodology, Investigation. **Ling Fu:** Investigation. **Fan Zhang:** Investigation. **Haiyan Liu:** Investigation. **Kai Wu:** Conceptualization. **Yanling Zhou:** Validation, Project administration, Methodology, Investigation. **Yuping Ning:** Conceptualization, Supervision, Writing – review & editing.

## Declaration of Competing Interest

The authors declare that they have no known competing financial interests or personal relationships that could have appeared to influence the work reported in this paper.

## Data Availability

The authors do not have permission to share data.
