# Supplementary material for: Baseline patterns of resting functional connectivity within posterior default-mode intranetwork associated with remission to antidepressants in major depressive disorder
Source: Neuroimage Clin. 2022 Oct 14;36:103230. doi: 10.1016/j.nicl.2022.103230 (PMC9668631; doi:10.1016/j.nicl.2022.103230)
Supplement: Supplementary data 1 [file mmc1.docx]

| **Serial numbers of discarded ICs** | **Identified Components** |
| --- | --- |
| Component 01 | physiological artifacts |
| Component 02 | cerebellum |
| Component 03 | physiological artifacts |
| Component 04 | physiological artifacts |
| Component 05 | physiological artifacts |
| Component 06 | physiological artifacts |
| Component 07 | physiological artifacts |
| Component 08 | physiological artifacts |
| Component 09 | physiological artifacts |
| Component 10 | physiological artifacts |
| Component 11 | cerebrospinal fluid |
| Component 12 | scalp |
| Component 13 | physiological artifacts |
| Component 14 | physiological artifacts |
| Component 15 | physiological artifacts |
| Component 16 | physiological artifacts |
| Component 17 | cerebrospinal fluid |
| Component 18 | physiological artifacts |
| Component 19 | physiological artifacts |
| Component 20 | physiological artifacts |
| Component 21 | physiological artifacts |
| Component 22 | physiological artifacts |
| Component 23 | cerebellum |
| Component 24 | scalp |
| Component 25 | physiological artifacts |
| Component 26 | physiological artifacts |
| Component 27 | physiological artifacts |
| Component 28 | overlap with cerebrospinal fluid |

**Table S1.** Twenty-eight discarded ICs


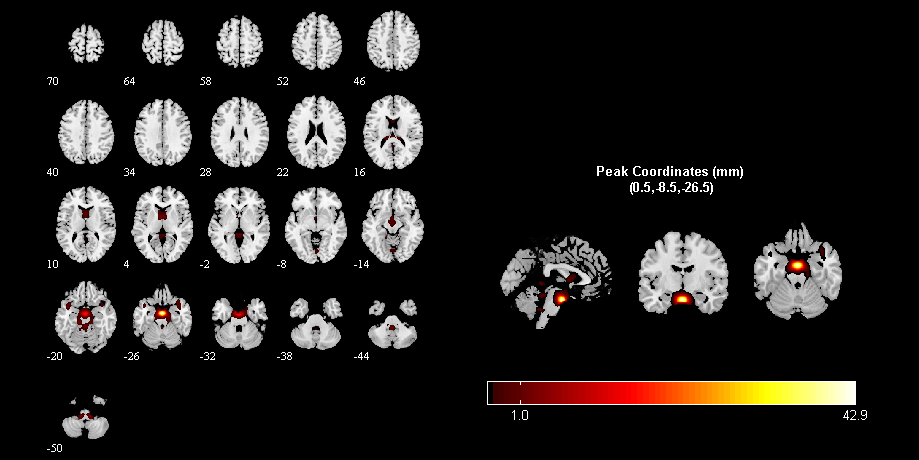
**Figure S1.** Component 01


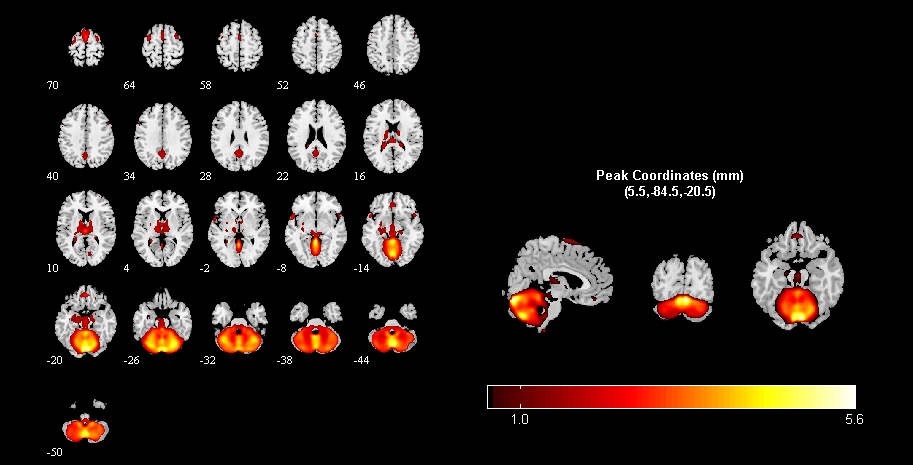


**Figure S2.** Component 02


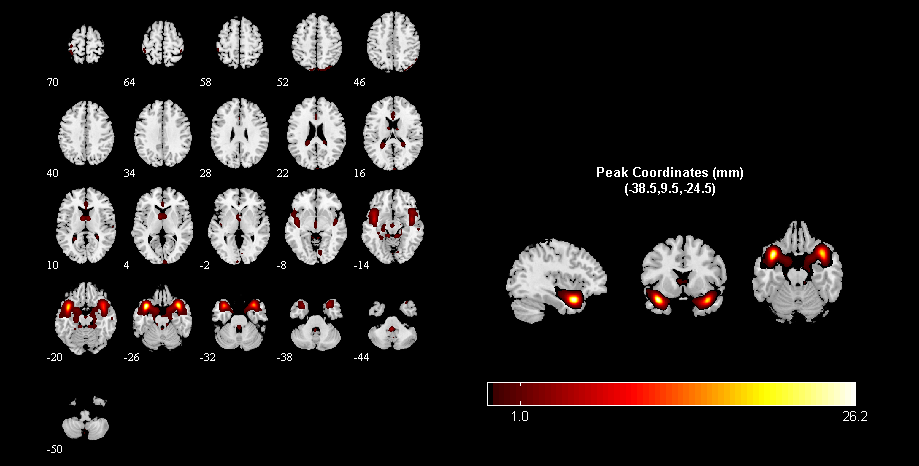
**Figure S3.** Component 03


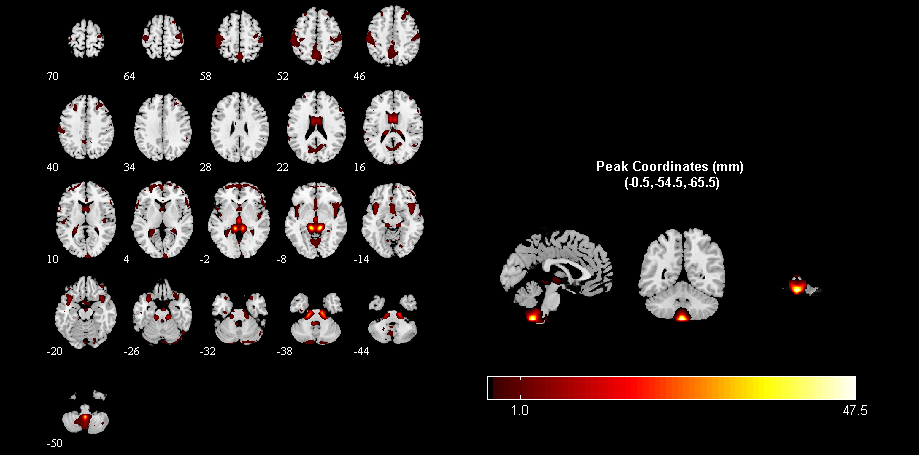


**Figure S4.** Component 04


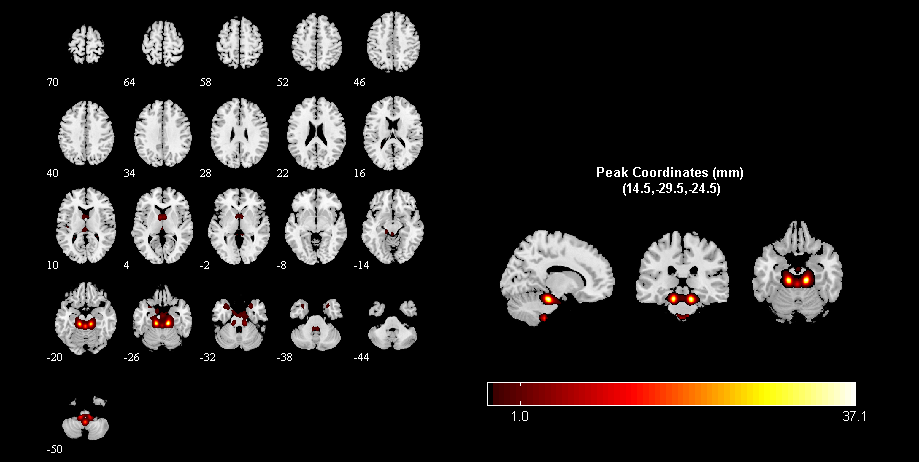


**Figure S5.** Component 05


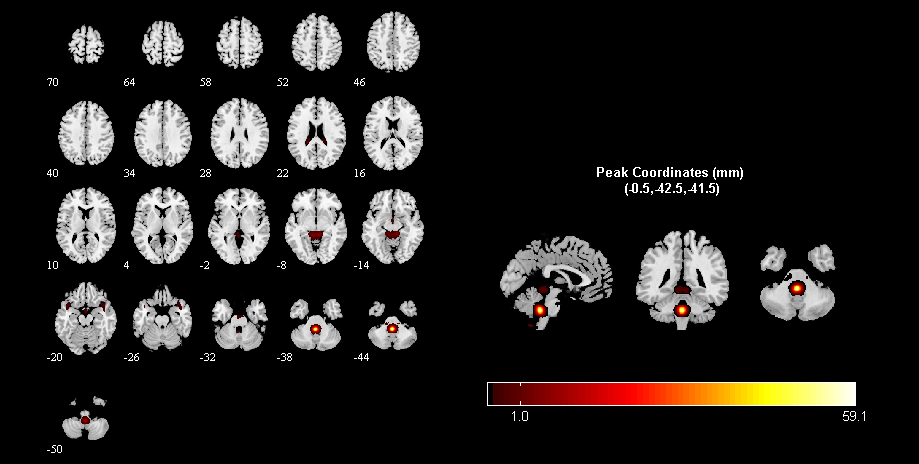


**Figure S6.** Component 06


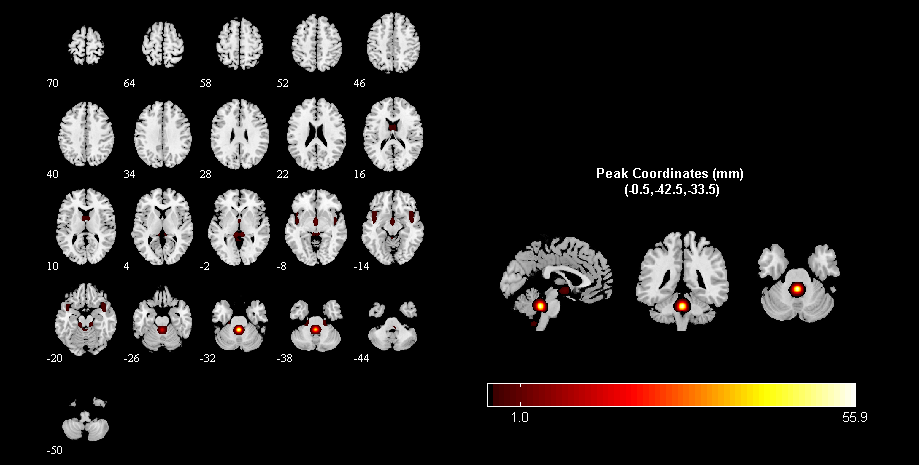


**Figure S7.** Component 07


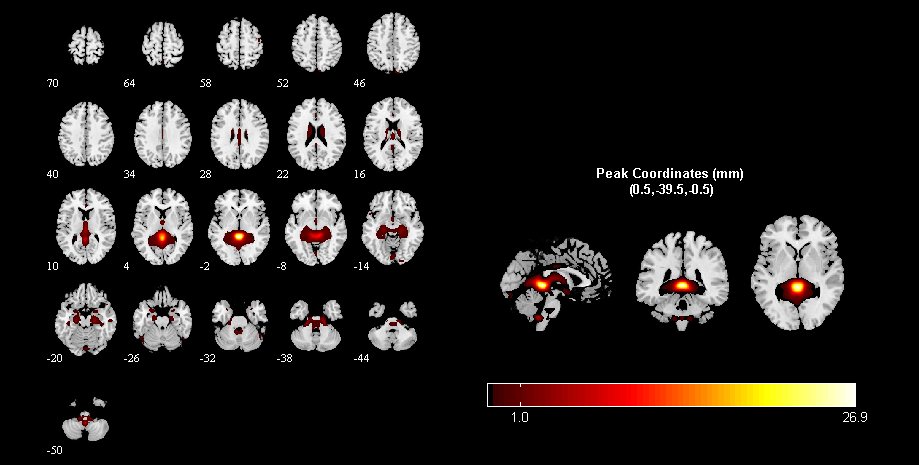


**Figure S8.** Component 08


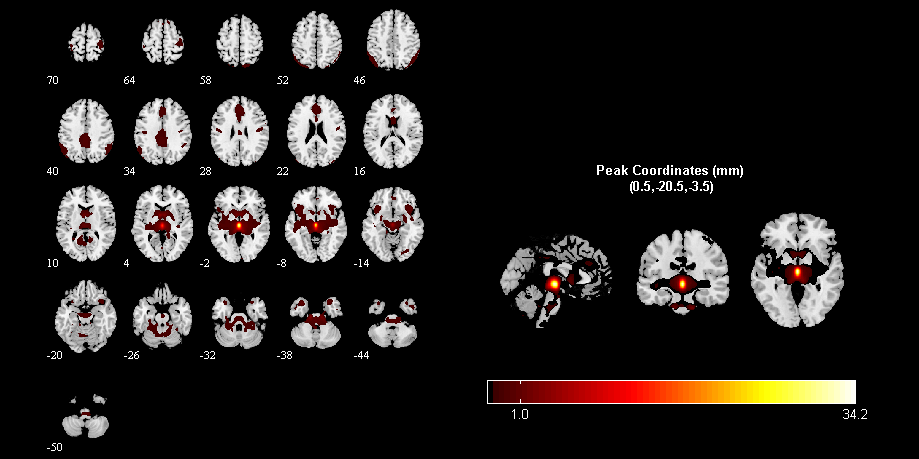


**Figure S9.** Component 09


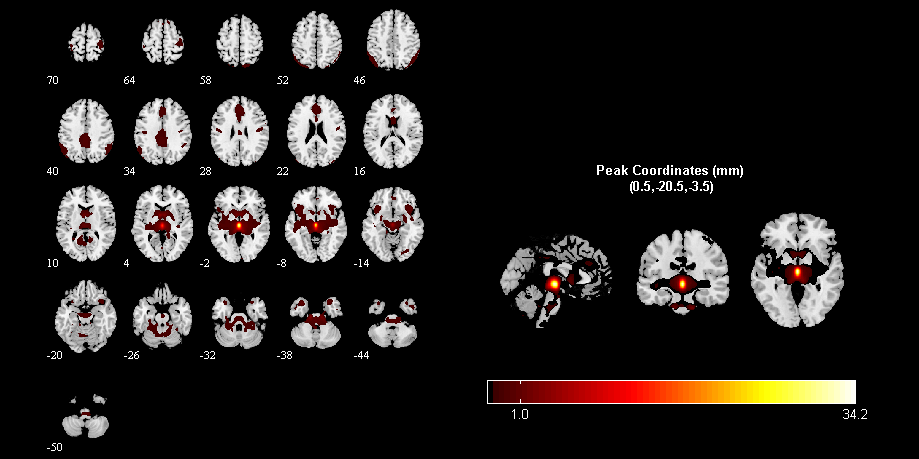


**Figure S10.** Component 10


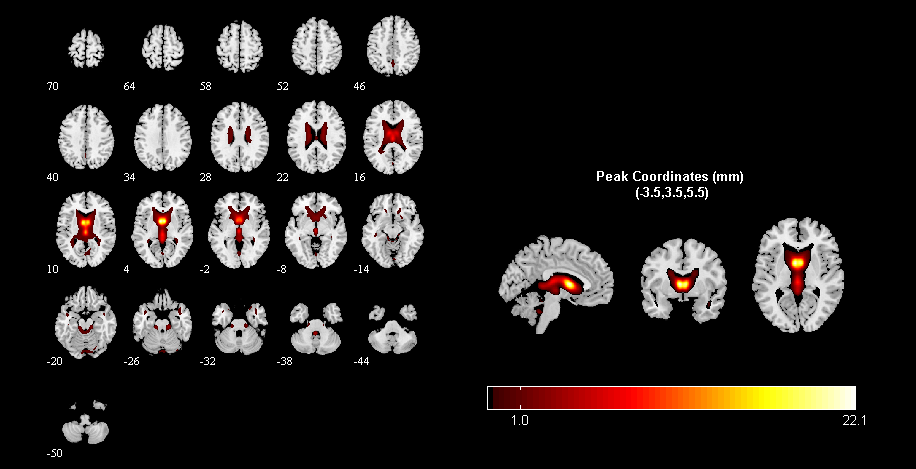


**Figure S11.** Component 11


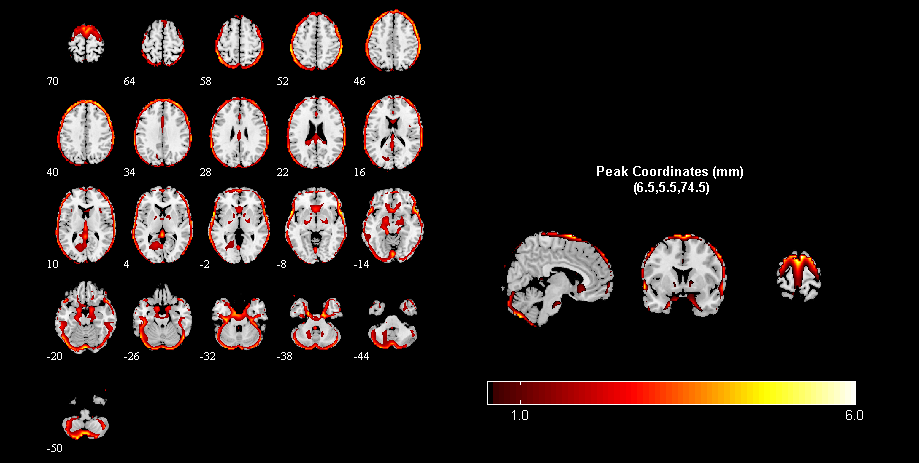
**Figure S12.** Component 12

**
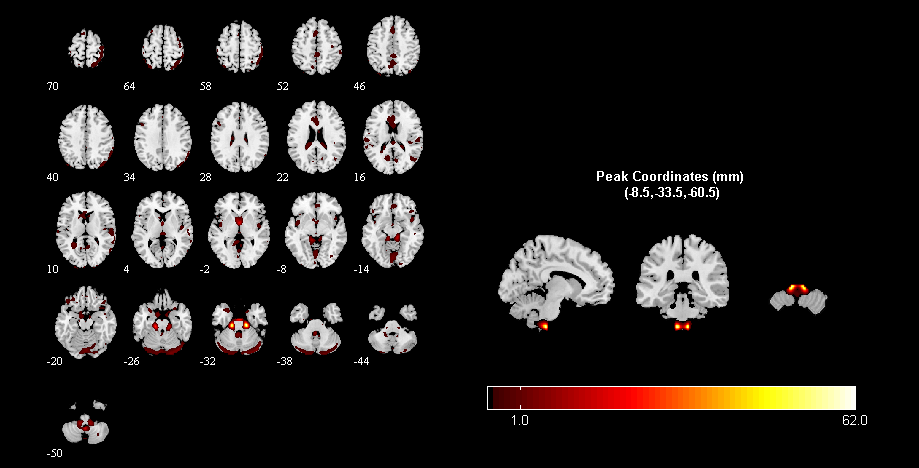
**

**Figure S13.** Component 13


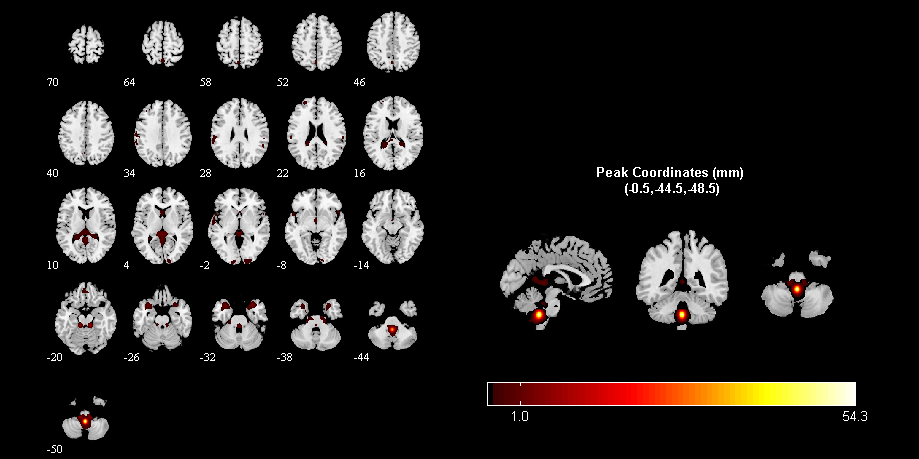


**Figure S14.** Component 14


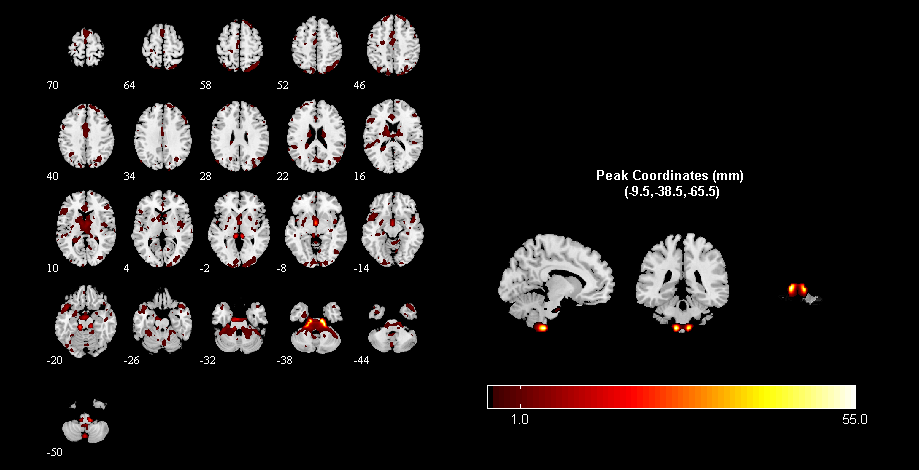


**Figure S15.** Component 15


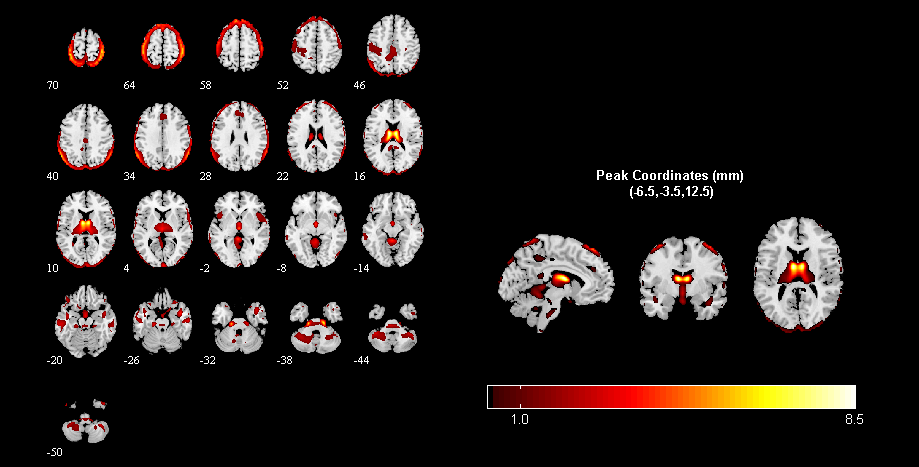


**Figure S16.** Component 16


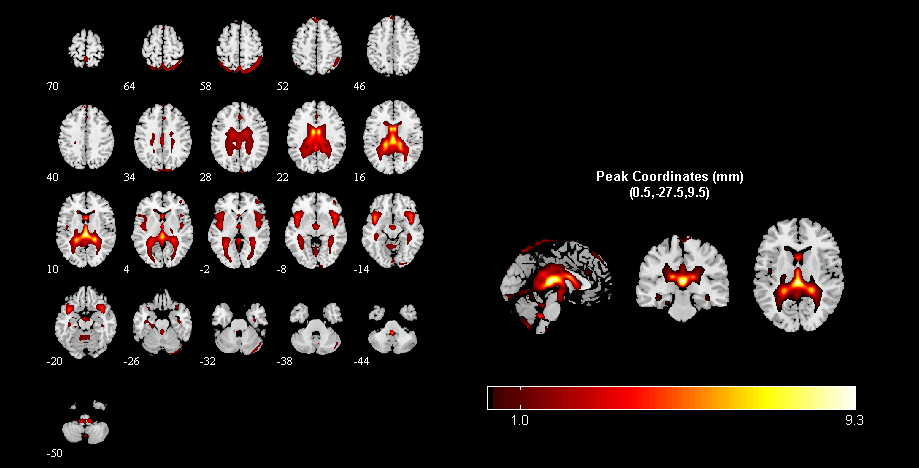


**Figure S17.** Component 17


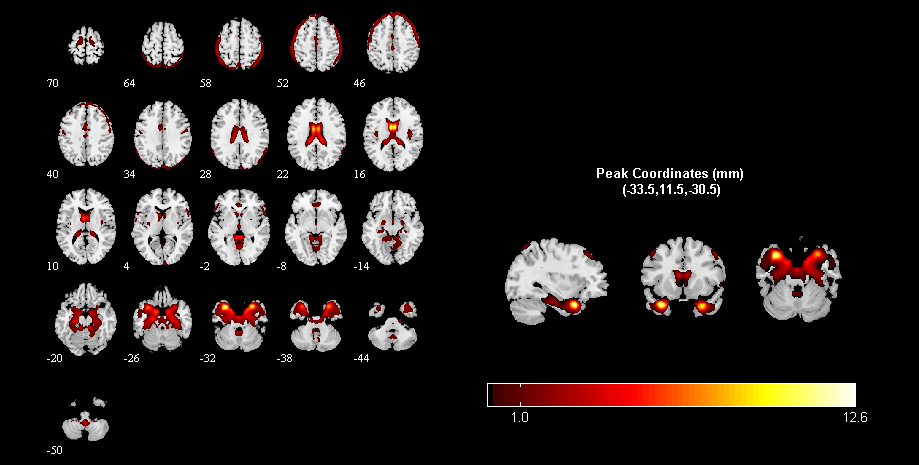


**Figure S18.** Component 18


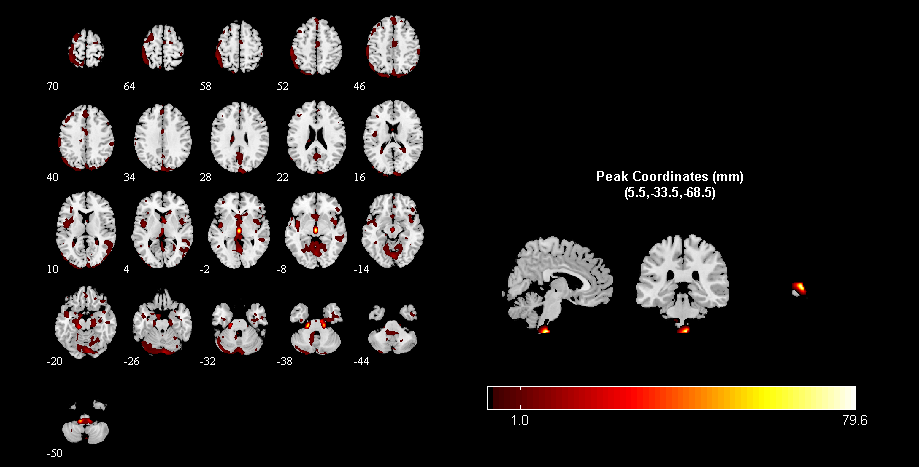


**Figure S19.** Component 19


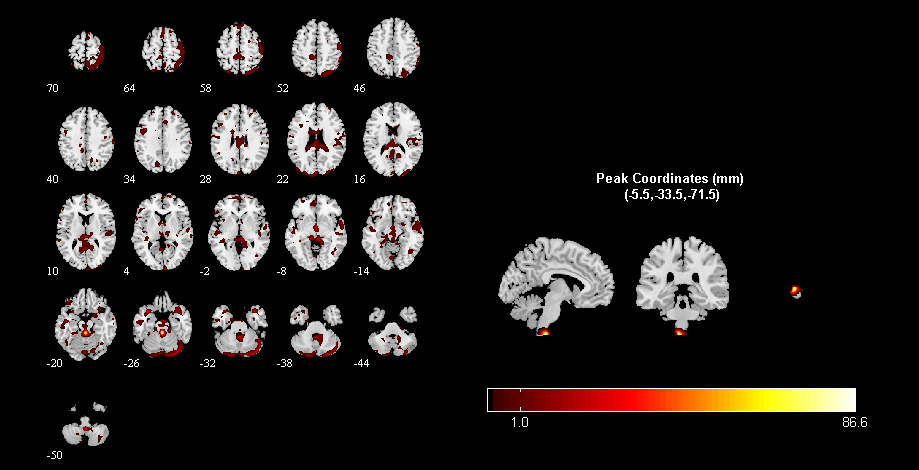


**Figure S20.** Component 20


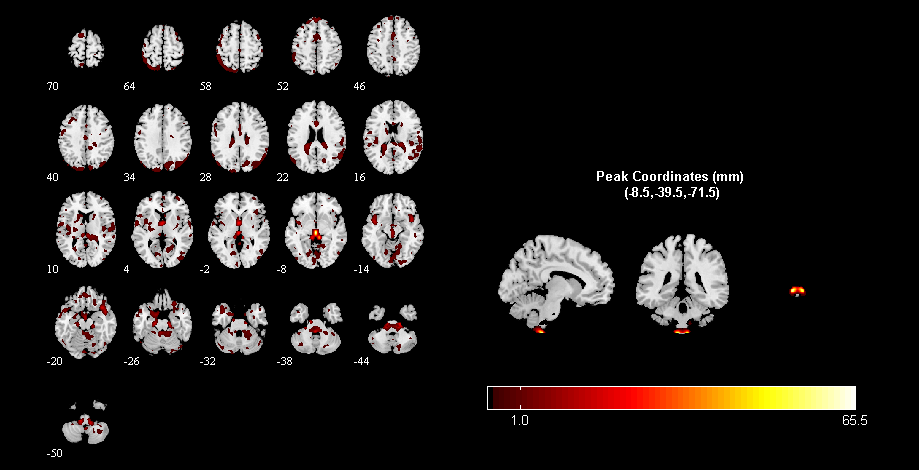


**Figure S21.** Component 21


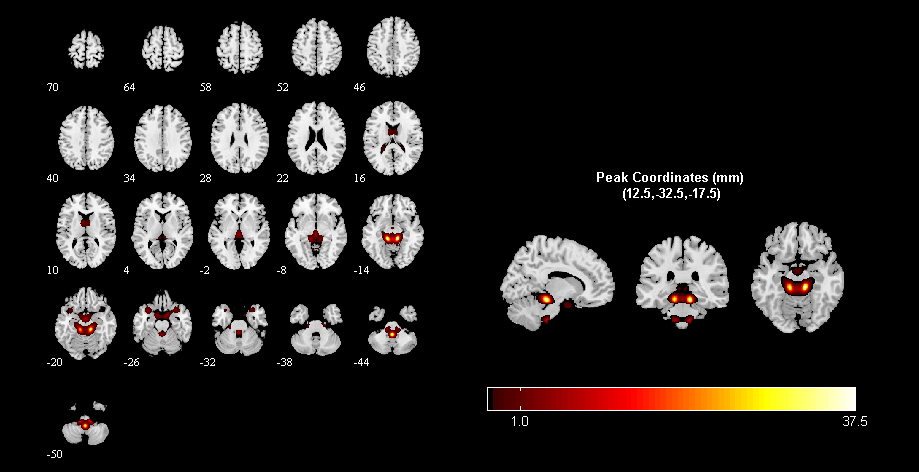


**Figure S22.** Component 22


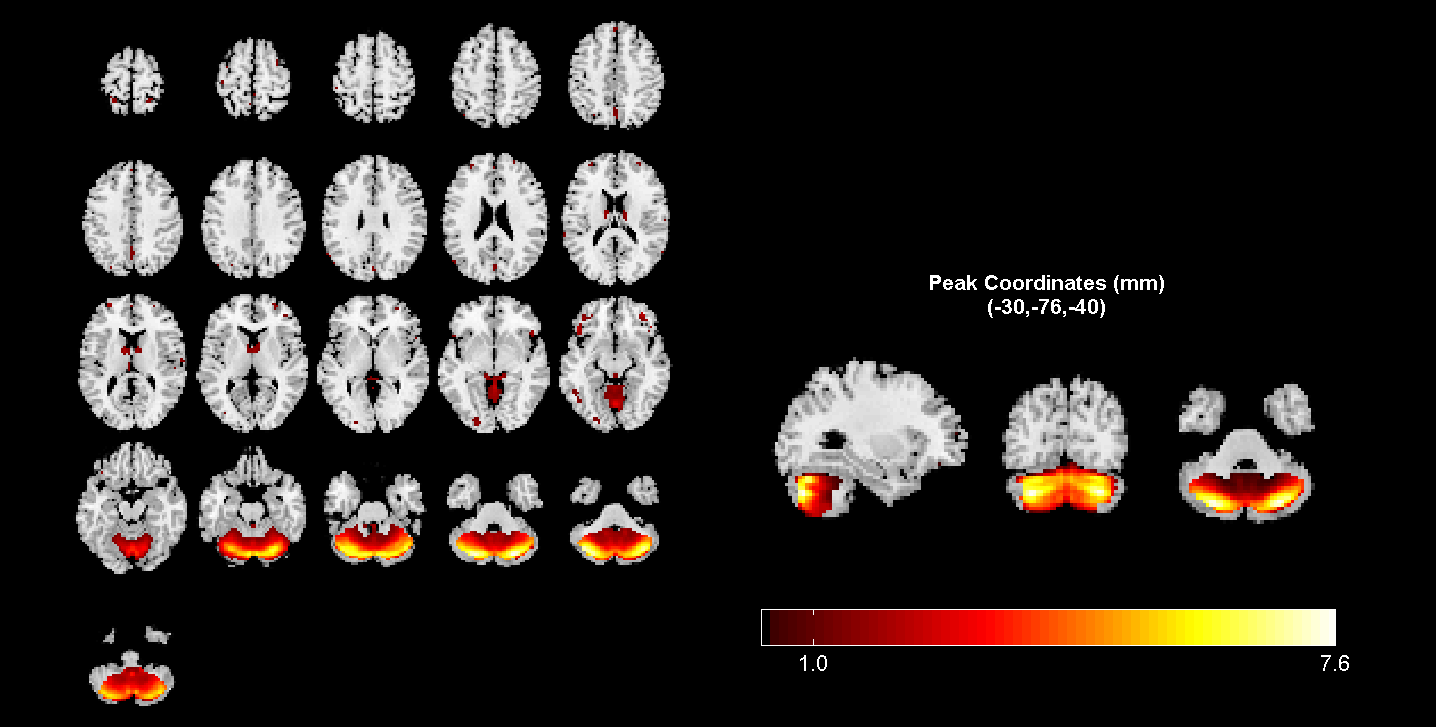


**Figure S23.** Component 23


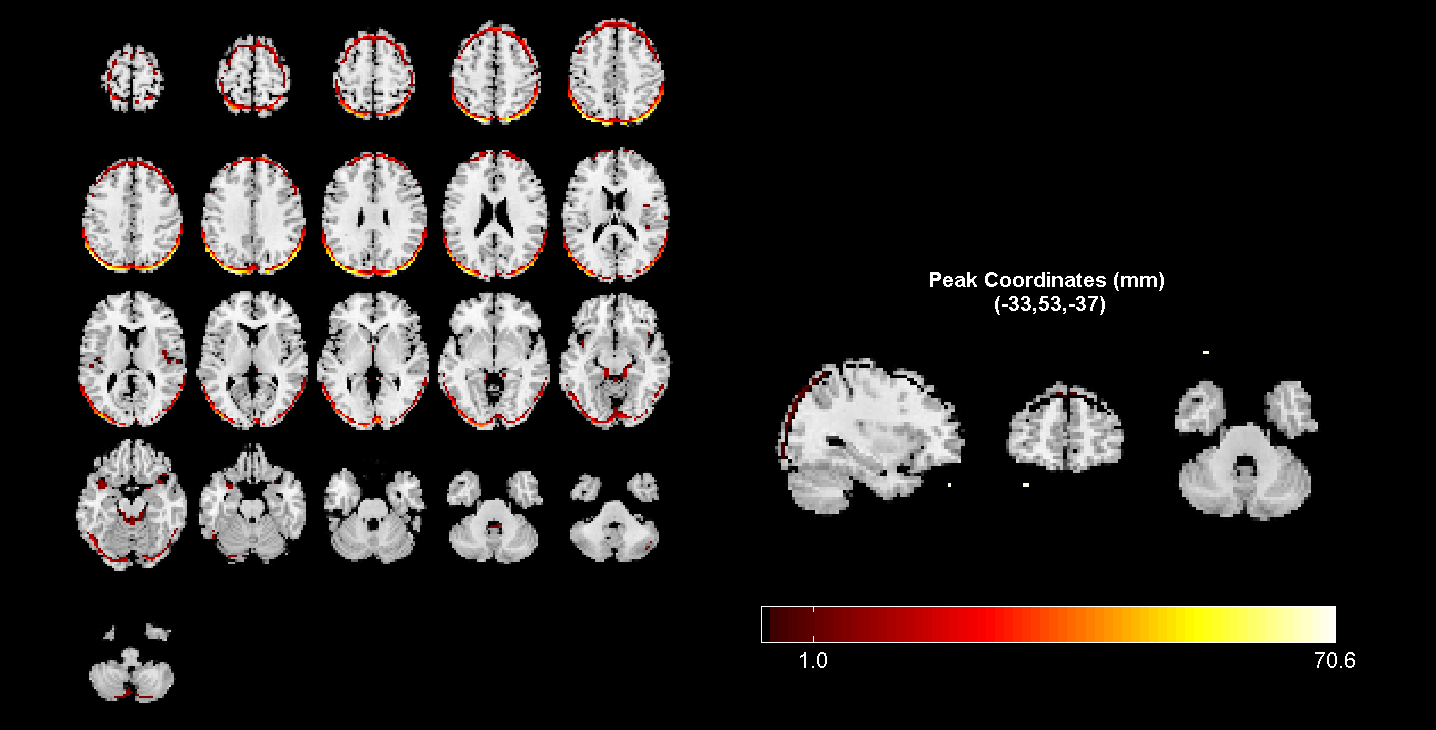
**Figure S24.** Component 24


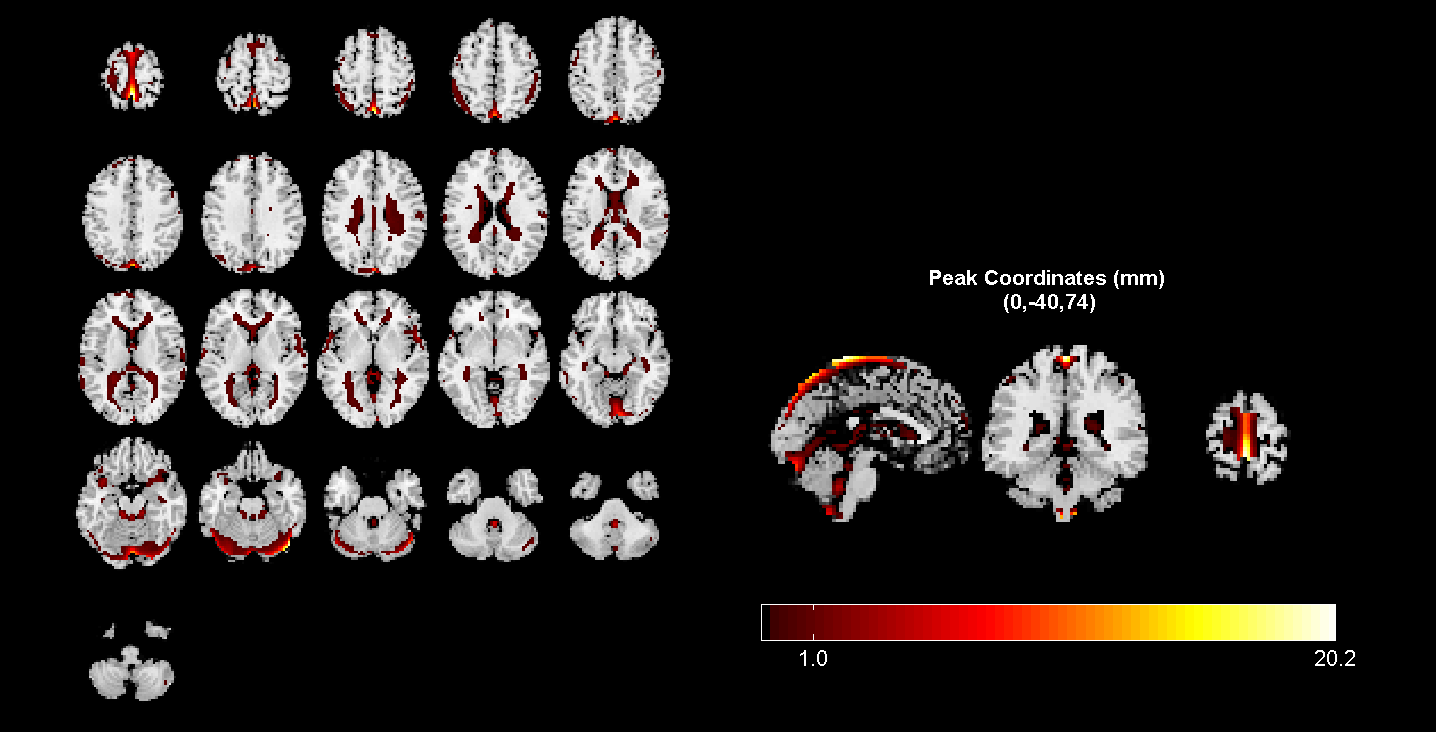


**Figure S25.** Component 25


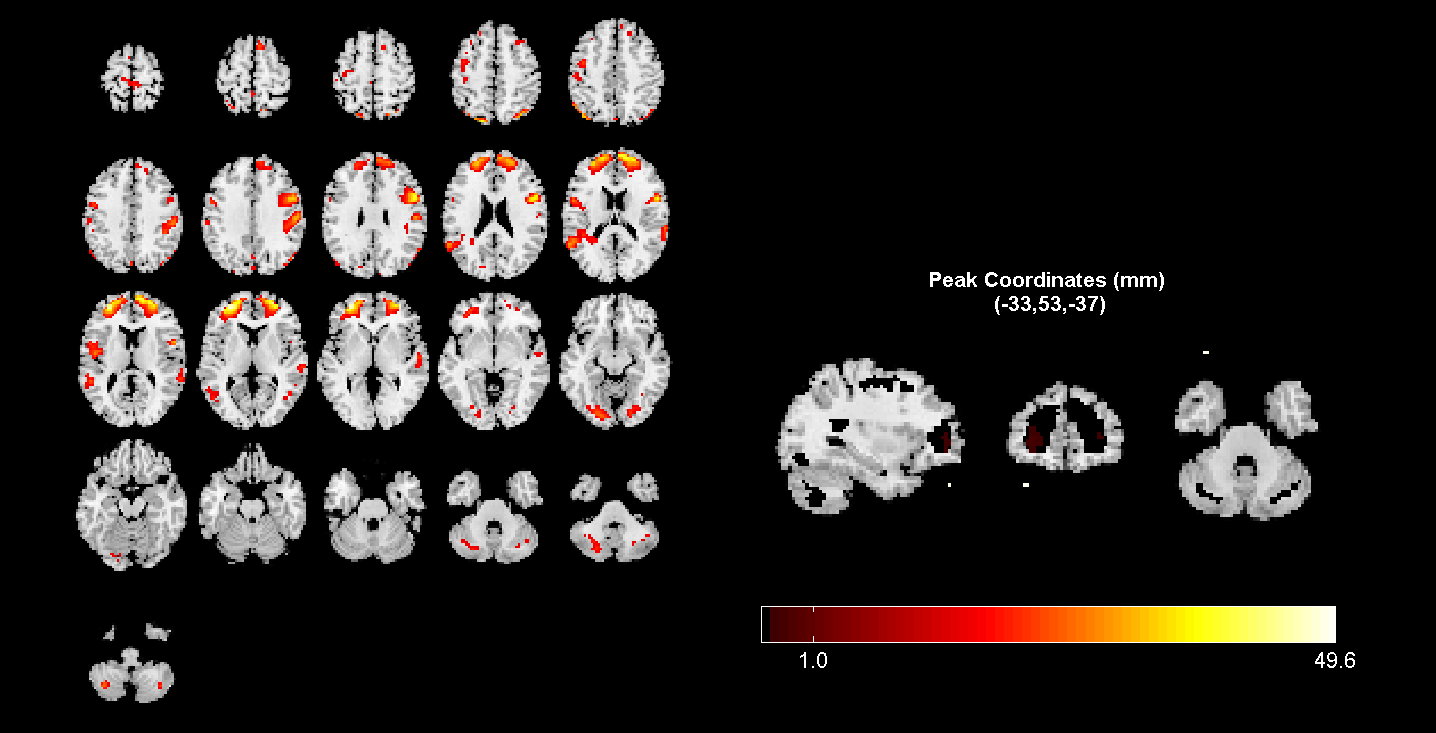


**Figure S26.** Component 26


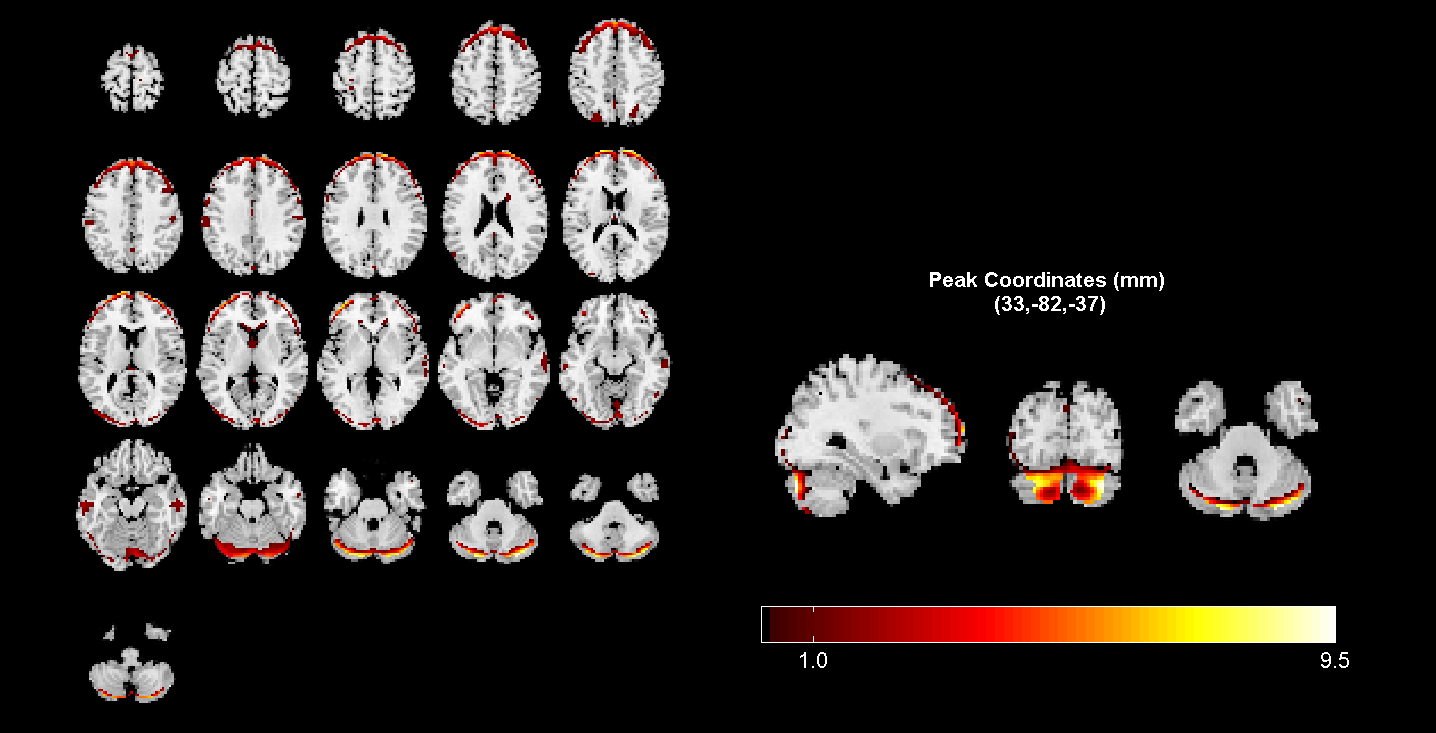
**Figure S27.** Component 27

**
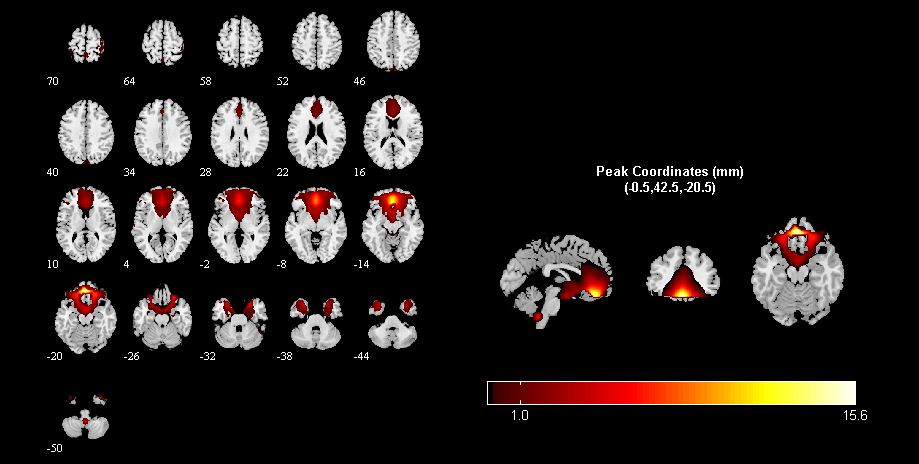
**

**Figure S28.** Component 28
